# Prostaglandin F2α agonist-induced suppression of 3T3-L1 cell adipogenesis affects spatial formation of extra-cellular matrix

**DOI:** 10.1038/s41598-020-64674-1

**Published:** 2020-05-14

**Authors:** Yosuke Ida, Fumihito Hikage, Kaku Itoh, Haruka Ida, Hiroshi Ohguro

**Affiliations:** Departments of Ophthalmology, Sapporo Medical University School of Medicine, Sapporo, Japan

**Keywords:** Clinical pharmacology, Glaucoma

## Abstract

To establish a deepening of the upper eyelid sulcus (DUES) model that can be induced by prostaglandin (PG) analogues, a three-dimension (3D) tissue culture was employed. Upon adipogenesis of the 3T3-L1 organoid, the effects of either Bimatoprost acid (BIM-A), or PGF2α were examined. During the adipogenesis, organoid size, lipid staining by BODIPY and expression of the extracellular matrix (ECM) by immunocytochemistry and/or quantitative PCR were employed. The size of the organoid increased remarkably during the adipogenesis, while such increases were significantly inhibited by the presence of PGF2α or BIM-A. BODIPY positive lipid-laden cells significantly increased during the adipogenesis, while in contrast they were greatly suppressed by the presence of PGF2α. Characteristic and spatial changes in ECM expressions observed upon adipogenesis were greatly modified by the presence of PGs. Our present study using a 3D tissue culture may be a suitable strategy toward understanding disease etiology of DUES.

## Introduction

Prostaglandin analogues (PGs) have powerful effects toward lowering intraocular pressures (IOP) by affecting prostanoid FP receptors. Moreover, because they also have few systemic side effects, they are widely used as a first-line drug for glaucoma^1^. The PGs group has shown similar mechanisms of IOP-lowering effects based on several meta-analysis studies^2–5^. Recently however, prostaglandin-associated periorbitopathy (PAP) has been associated as a local side effect occurring during long periods of PGs clinical use. The most frequent PAP is deepening of the upper eyelid sulcus (DUES)^6–9^, but elongation of eyelashes, hyperpigmentation of the skin, and changes in iris color have also been reported^10^. A recent study, utilizing magnetic resonance imaging, reported that long-term application of PGs caused reduction of orbital adipose tissues^11^. The adipocyte densities of eyelids in the patients using travoprost and Bimatoprost (BIM) were significantly higher than those of untreated contralateral sides^7^. These observations suggested that PGs such as travoprost or BIM caused a decrease in mean adipocyte volume and an increase in adipocyte density in eyelids.

Among PGs, DUES by BIM was to first to be reported^10,12^, and it occurred more frequently than latanoprost. It was reported that by switching latanoprost to BIM, the incidence of DUES was approximately 60%^12^, and in turn, such high frequency of DUES by BIM was reduced by switching back to latanoprost^10^. Thus, BIM and travoprost have more risk for DUES than latanoprost. Based upon the above evidence of different DUES frequency and similar IOP lowering effects among PGs, other unknown mechanisms are likely involved in etiology of DUES by PGs. However, further mechanisms causing DUES by PGs have yet to be fully elucidated.

Adipocytes are known to be involved in the control of energy homeostasis by regulating the fat storage and mobilization of free fatty acids in response to a variety of nutritional and hormonal conditions^13,14^. Thus, it is well known that adipose tissue volumes can be altered under various conditions. Adipogenesis requires the differentiation of preadipocytes into mature adipocytes through transcriptional programs regulating specific adipogenic genes including C/EBPβ/δ and PPARγ^15^. To reach PPARγ induced maturity of the adipocyte differentiation there are known to be modulated by some stimulators including PGs, insulin-like growth factor 1 (IGF-1), insulin, cAMP, triiodothyronine, macrophage colony stimulating factor, fatty acids, and glucocorticoids^16,17^, as well as inhibitors including glycoproteins, transforming growth factor-β (TGF-β), inflammatory cytokines and growth hormone^18^. Among these factors, PGs is well known to be one of the factors that affect the adipogenesis, since PGE2 and PGF2α suppress the differentiation of adipocytes through EP4 and FP receptors, respectively^19^. In fact, adipogenesis of 3T3-L1 (preadipocyte cell line) is significantly inhibited by treatment with PGs including PGF2α or fluprostenol, a prostanoid FP2 receptor agonist^20^.

Based upon the assumption adipocytes should spread toward three-dimensional (3D) spaces within the body during its differentiation, a 3D cell culture system should be more desirable than conventional two-dimension (2D) cell culture methods in this research field. However, little research has been conducted using a 3D cell culture system because of the difficulties concerning their techniques. Recently, the 3D tissue culture has emerged as a useful approach for modeling human diseases^21^. Theoretically this technique offers the potential for recapitulating cell-cell and cell-extracellular matrix (ECM) interactions that have not been possible in standard 2D culture models^22^. Most recently, to understand the molecular etiology of thyroid-associated orbitopathy, our group utilized the 3D cell culture of human orbital fibroblasts, and found that hypoxia-inducible factor (HIF) 2α (HIF2A), but not its paralog HIF1A, accelerates ECM deposition by inducing a collagen-cross-linking enzyme, lysyl oxidase (LOX). Taken together, to elucidate what kinds of mechanisms within the adipogenesis are involved in the etiology of DUES by PGs, the recently developed 3D cell culture system should be more suitable.

Therefore, in the current study, using this 3D cell culture technique, we replicated pathogenic tissue remodeling of DUES by culturing 3T3-L1 cells and analyzed the effects of PGs on adipocyte volume and ECM expression during the adipogenesis.

## Results

**Figure 1:**
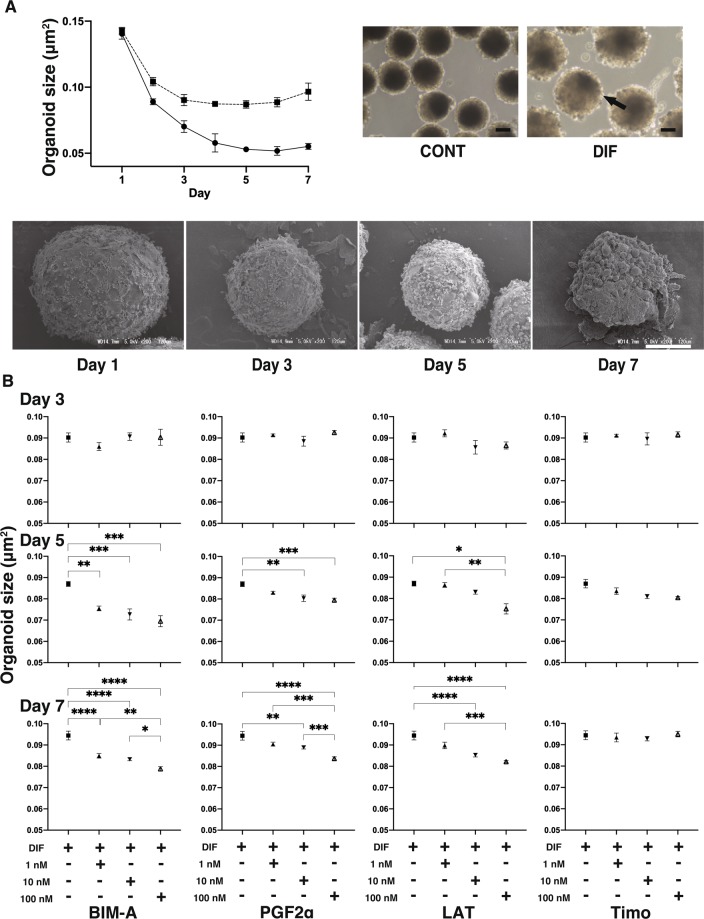
Effects of PGs on sizes of 3T3-L1 3D organoids during adipogenesis (**A**). The mean sizes of 3D organoids of 3T3-L1 preadipocytes as a control (CONT, closed circles) and their differentiation (DIF, closed squares) were plotted in the left upper panel from Day 1 through Day 7. Representative images of phase contrast microscopy (an arrow indicates lipid-laden adipocytes, scale bar: 100 μm) of 3D CONT and DIF 3T3-L1 organoids on Day 7 are shown in the upper right panel. Those of electron microscopy of CONT 3T3-L1 organoids on Day 1, 3, 5 and 7 are shown in the lower panels. (**B**) To study the effects of PGs on means sizes of 3D DIF 3T3-L1 organoids, those were plotted as above in the presence of Bimatoprost free acid (BIM-A), Prostaglandin F2α (PGF2α) or latanoprost (LAT) at concentrations of 1, 10 or 100 nM on Day 3, 5 or 7. As a control, timolol (Timo) was used as above. In the presence of PGs, the mean sizes of organoids became significantly smaller after Day 5 in a concentration dependent manner. Among PGs, the strongest suppression was observed in BIM-A, but similar effects were noticed in PGF2α and LAT. In contrast, no effects were detected by Timo. All experiments were performed in triplicate using fresh preparations consisting of 16 organoids each. Data are presented as arithmetic means ± standard error of the mean (SEM). *P < 0.05, **P < 0.01, ***P < 0.005, ****P < 0.001 (ANOVA followed by a Tukey’s multiple comparison test).

To elucidate the pathological mechanisms of PGs toward DUES *in vivo*, 3D cell cultures of 3T3-L1 cells were used in the present study. Uniform round-shape spheroidal organoids were generated from 20,000 3T3-L1 preadipocytes (CONT), and under adipogenic differentiation during the 7 day culture (DIF), the size of the organoids significantly increased (Fig. 1A, upper panels). During the course of the 3D culture, premature organoids grew into their matured form with a number of lipids marked by arrows (Fig. 1A, lower panel). As shown in Fig. 1B, during the course of the culture adipogenesis-induced enhancement of the organoid sizes was apparently less in the presence of PGs including Bimatoprost free acid (BIM-A), Prostaglandin F2α (PGF2α) or latanoprost in a concentration dependent manner (1–100 nM). In contrast, no significant effects were observed by the presence of timolol (1–100 nM). Among the PGs, BIM-A caused the stronger effects, but PGF2α and latanoprost induced almost similar effects. Therefore, both BIM-A and PGF2α were used as PGs in subsequent studies.

**Figure 2:**
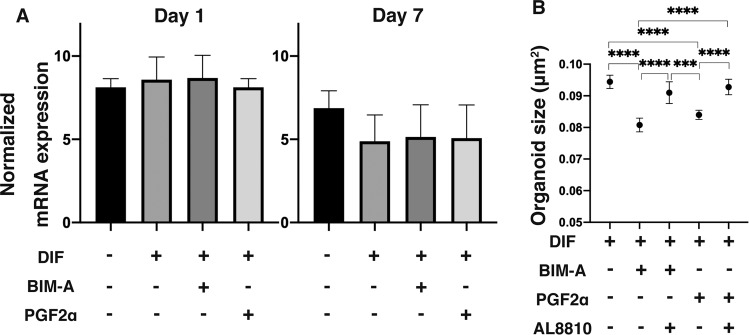
mRNA expression of PG FP receptors, and effects of its antagonist toward sizes of DIF 3D organoids (**A**) On Day 1 and 7, 3D culture organoids of 3T3-L1 preadipocytes as the control (CONT) and their differentiation in the absence (DIF) or presence of 100 nM Bimatoprost free acid (BIM-A) or 100 nM Prostaglandin F2α (PGF2α) were subjected to qPCR analysis to estimate mRNA expression of prostaglandin FP receptors. The expressions of prostaglandin FP receptors were detected both on Day 1 and 7. By Day 7, the expressions of prostaglandin FP receptors had increased relatively in the CONT group. (**B**) To study the effects of AL8810, a specific antagonist of FP receptors, the mean sizes of DIF 3D organoids in the presence of PGs and/or AL8810 on Day 7 were plotted. Effects of PGs (BIM-A and PGF2α) that caused significant reduction of the mean sizes of DIF 3D organoids as noted above were diminished by the presence of AL8810, a specific antagonist of FP receptors. Data are presented as arithmetic means ± standard error of the mean (SEM). ***P < 0.005, ****P < 0.001 (ANOVA followed by a Tukey’s multiple comparison test).

As shown in Fig. 2A, mRNA expressions of FP receptors were observed from Day 1 of the 3D culture and were slightly suppressed upon PGs administration through Day 7, suggesting that the effects by PGs were mediated through FP receptors. Furthermore, a specific inhibitor of FP receptors, AL8810, strongly blocked PGs-induced size changes in the 3D organoids (Fig. 2B). As above, these observations suggest that effects of PGs toward 3D organoids should be mediated through FP receptors.

**Figure 3:**
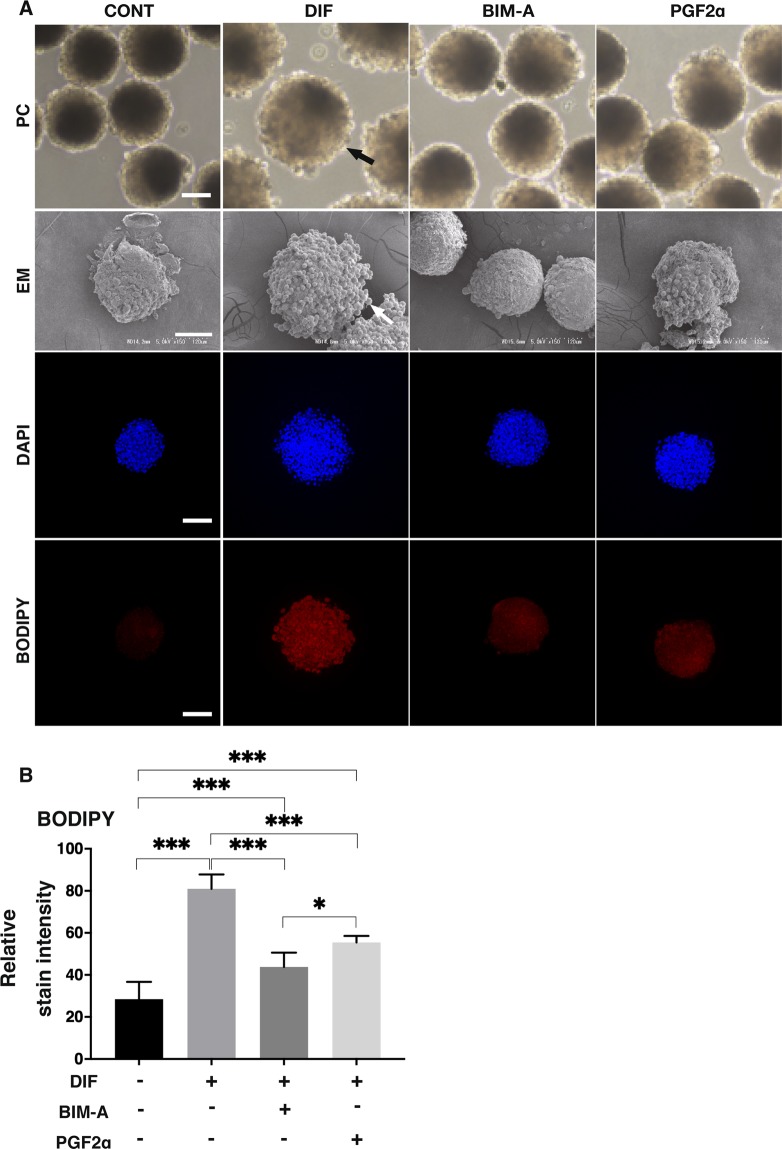
Representative phase contrast microscope or electron microscope images of 3D organoids, and their confocal images of lipid staining (BODIPY) under several conditions. On Day 7, images of 3D organoids under several conditions were immune-stained by DAPI (blue) and BODIPY (red) and observed through phase contrast microscopy (PC) or electron microscopy (EM) (**A**). Their staining intensities by BODIPY were plotted (**B**). Only a faint staining by BODIPY was observed in the organoids of 3T3-L1 preadipocytes (CONT). Through the adipogenic differentiation, their staining intensities were significantly enhanced (DIF), but such enhancements were significantly suppressed by the presence of 100 nM BIM-A and PGF2α. All experiments were performed in duplicate using fresh preparations consisting of 10 organoids each. Data are presented as arithmetic means ± standard error of the mean (SEM). *P < 0.05, ***P < 0.005 (ANOVA followed by a Tukey’s multiple comparison test).

During the adipogenic differentiations, a number of lipid-laden adipocytes were observed in the DIF as compared to the CONT (marked by an arrow in Fig. 3A PC and EM). Such lipid-laden adipocytes also decreased in the presence of 100 nM BIM-A or PGF2α. To study this further, lipid staining of the 3D organoids was performed by BODIPY (Fig. 3A BODIPY and B). Only a faint staining was observed in the CONT. In contrast, its staining intensities were significantly increased in the DIF, but seemingly less in the presence of BIM-A or PGF2α (Fig. 3B).

**Figure 4:**
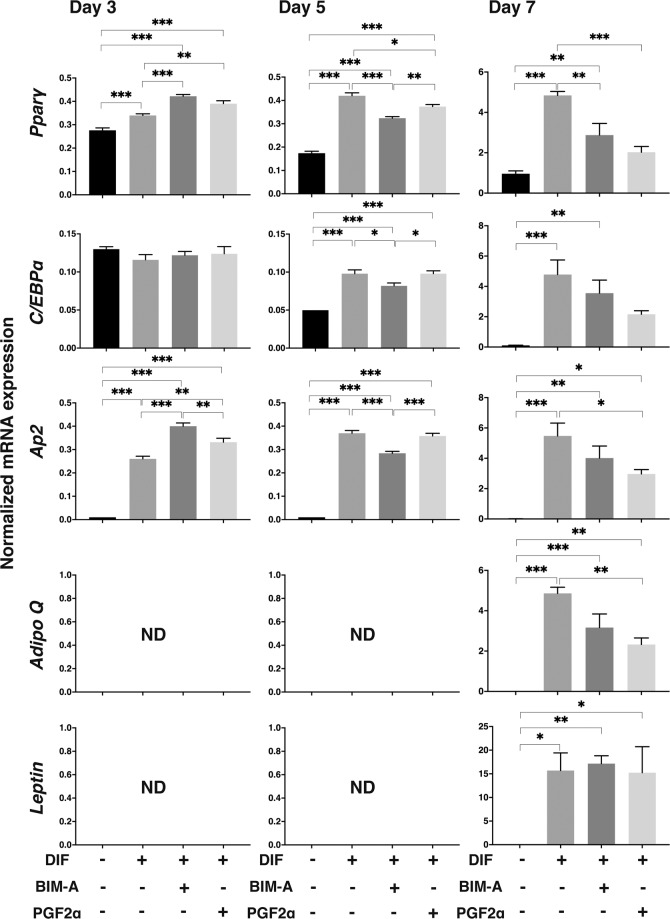
mRNAs expressions of adipogenesis related genes in cluding *Pparγ*, *C/EBPa, AP2, Adipo Q, and Leptin* under several conditions. At Day 3, 5 and 7, 3D culture organoids of 3T3-L1 preadipocytes as the control (CONT) and their differentiation in the absence (DIF) or presence of 100 nM Bimatoprost free acid (BIM-A) or 100 nM Prostaglandin F2α (PGF2α) were subjected to qPCR analysis and plotted to estimate mRNA expression of adipogenesis related genes including *Pparγ*, *C/EBPa, AP2, Adipo Q, and Leptin*. Those expressions of *Pparγ*, *C/EBPa, AP2, Adipo Q*, *or Leptin* were significantly increased during the adipogenetic differentiation as compare to CONT. Among these those of *Pparγ*, *C/EBPa, AP2*, and *Adipo Q* were marked suppressed by BIM-A and PGF2α by Day 7. All experiments were performed in duplicate using fresh preparations consisted of 5 organoids each. Data are presented as arithmetic means ± standard error of the mean (SEM). * *P* < 0.05, ** *P* < 0.01, *** *P* < 0.005 (ANOVA followed by a Tukey’s multiple comparison test). ND: not detected.

To elucidate what kinds of mechanisms are involved in PG induced effects of adipogenesis, mRNA expressions of major adipogenesis related genes including *Pparγ*, *C/EBPa, AP2, Adipo Q* and *Leptin* were investigated. As shown in Fig. 4, those of *Pparγ, C/EBPa, AP2* and *Adipo Q* increased significantly upon adipogenesis but were markedly suppressed by the presence of BIM-A or PGF2α on Day 7. Those of *Leptin* increased also during adipogenesis but were not altered by the presence of PGs.

**Figure 5:**
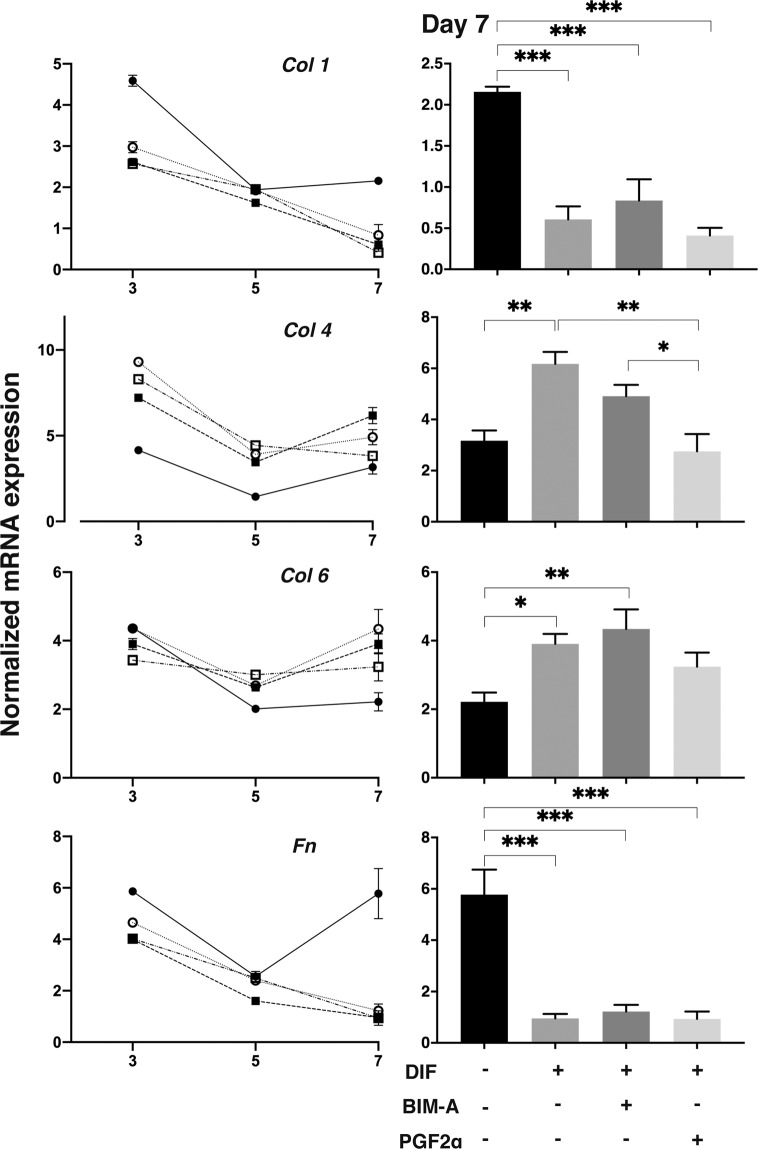
qPCR analyses of gene expressions of ECMs in 3D 3T3-L1 organoids. At Day 3, 5 and 7, 3D culture organoids of 3T3-L1 preadipocytes as the control (CONT, closed circles) and their differentiation in the absence (DIF, closed squares) or presence of 100 nM Bimatoprost free acid (BIM-A, open circles) or 100 nM Prostaglandin F2α (PGF2α, open squares) were subjected to qPCR analysis to estimate mRNA expression of ECMs (COL 1: collagen 1, COL 4: collagen 4, COL 6: collagen 6, FN: fibronectin). Fluctuations of the expressions during the period were plotted in left graphs. These mRNA expressions at Day 7 among conditions were also shown in right graphs with statistical analysis in right graphs. All experiments were performed in duplicate using fresh preparations consisted of 5 organoids each. Data are presented as arithmetic means ± standard error of the mean (SEM). * *P* < 0.05, ** *P* < 0.01, *** *P* < 0.005 (ANOVA followed by a Tukey’s multiple comparison test).

Next, to elucidate effects of BIM-A and PGF2α on the gene expression of the ECM during the adipogenesis, those of major ECM in the 3D organoids including collagen (COL) 1, COL 4, COL 6, and fibronectin (FN) were examined. As shown in Fig. 5, during the early phase of the 3D culture at Day 3 the levels of mRNA expressions of the CONT were significantly higher in COL 1 and FN, less in COL 4, and almost comparable as compared to the DIF in the absence or presence of PGs. Then these expressions of the ECMs once decreased during the middle phase (Day 5) as compared to their initial levels (Day 3) of all conditions tested. During the later phase of the culture (Day 5 to 7) those of COL 1 and FN increased in the CONT, but those further decreased upon adipogenesis in the presence or absence of PGs. In terms of COL 4 and COL 6 during the later phase, the mRNA expressions increased in both CONT and DIF, but levels of their increase were much higher in the DIF. Such effects of the DIF were less in the presence of PGF2α, but not in BIM-A. Thus at Day 7, as compare to the CONT (Fig. 5 right panels), 1) the DIF of COL 1 and FN were significantly lower in the presence or absence of PGs, and 2) the DIF of COL 4 or COL 6 significantly higher in the presence or absence of BIM-A.

**Figure 6:**
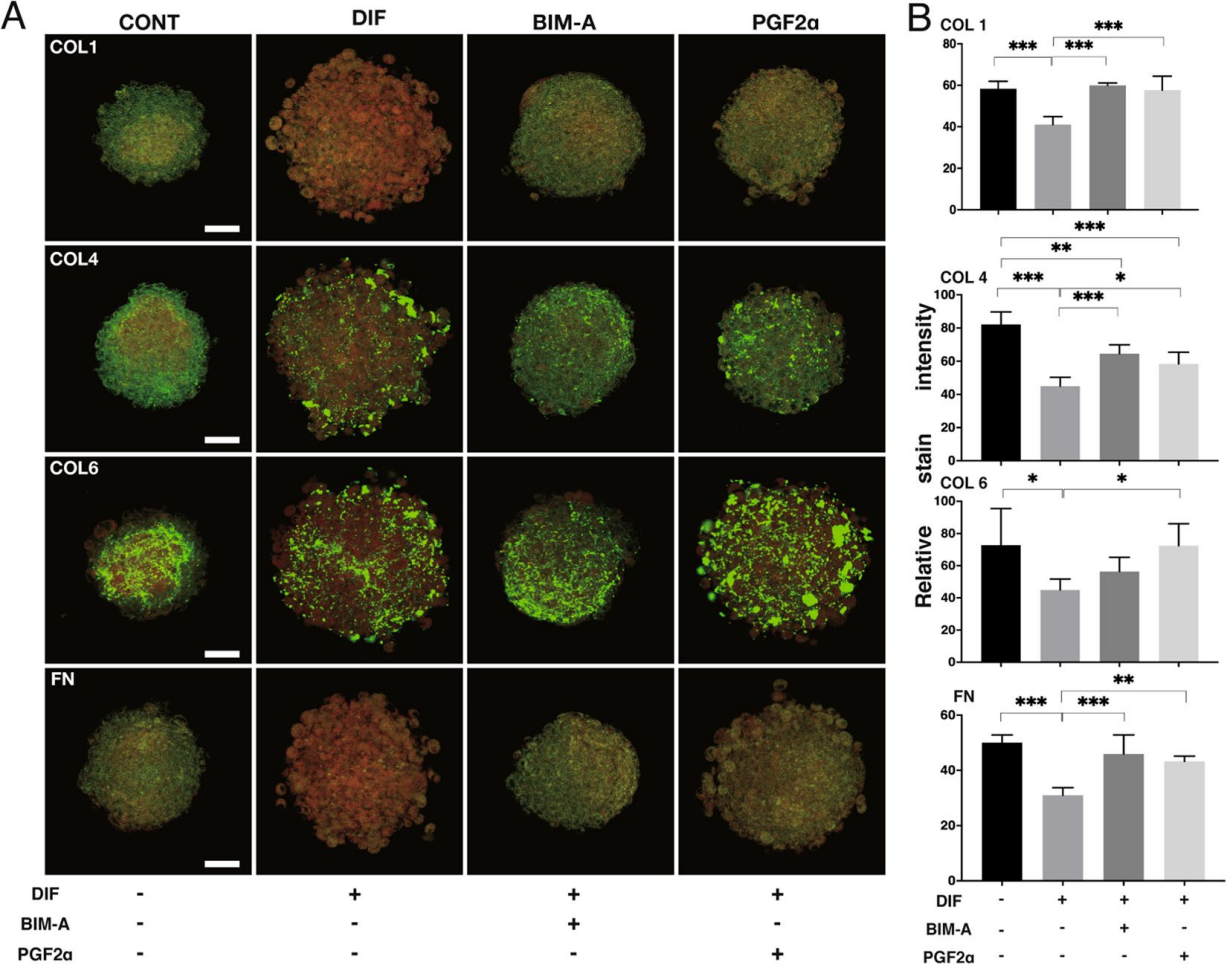
Confocal images of expressions of ECMs in 3D 3T3-L1 organoids under several conditions. A: At Day 7, 3D culture organoids of 3T3-L1 preadipocytes as the control (CONT) and their differentiation in the absence (DIF) or presence of 100 nM Bimatoprost free acid (BIM-A) or 100 nM Prostaglandin F2α (PGF2α) were immunostained by specific antibodies of ECMs including collagen 1 (COL 1), collagen 4 (COL 4), collagen 6 (COL6), or fibronectin (FN) designated by green in conjunction with BODIPY lipid staining designated by red. Scale bar: 100 μm. B: Staining intensities of the ECMs of the organoids stained as above were plotted in B. All experiments were performed in duplicate using fresh preparations consisted of 5 organoids each. Data are presented as arithmetic means ± standard error of the mean (SEM). * *P* < 0.05, ** *P* < 0.01, *** *P* < 0.005 (ANOVA followed by a Tukey’s multiple comparison test).

**Figure 7:**
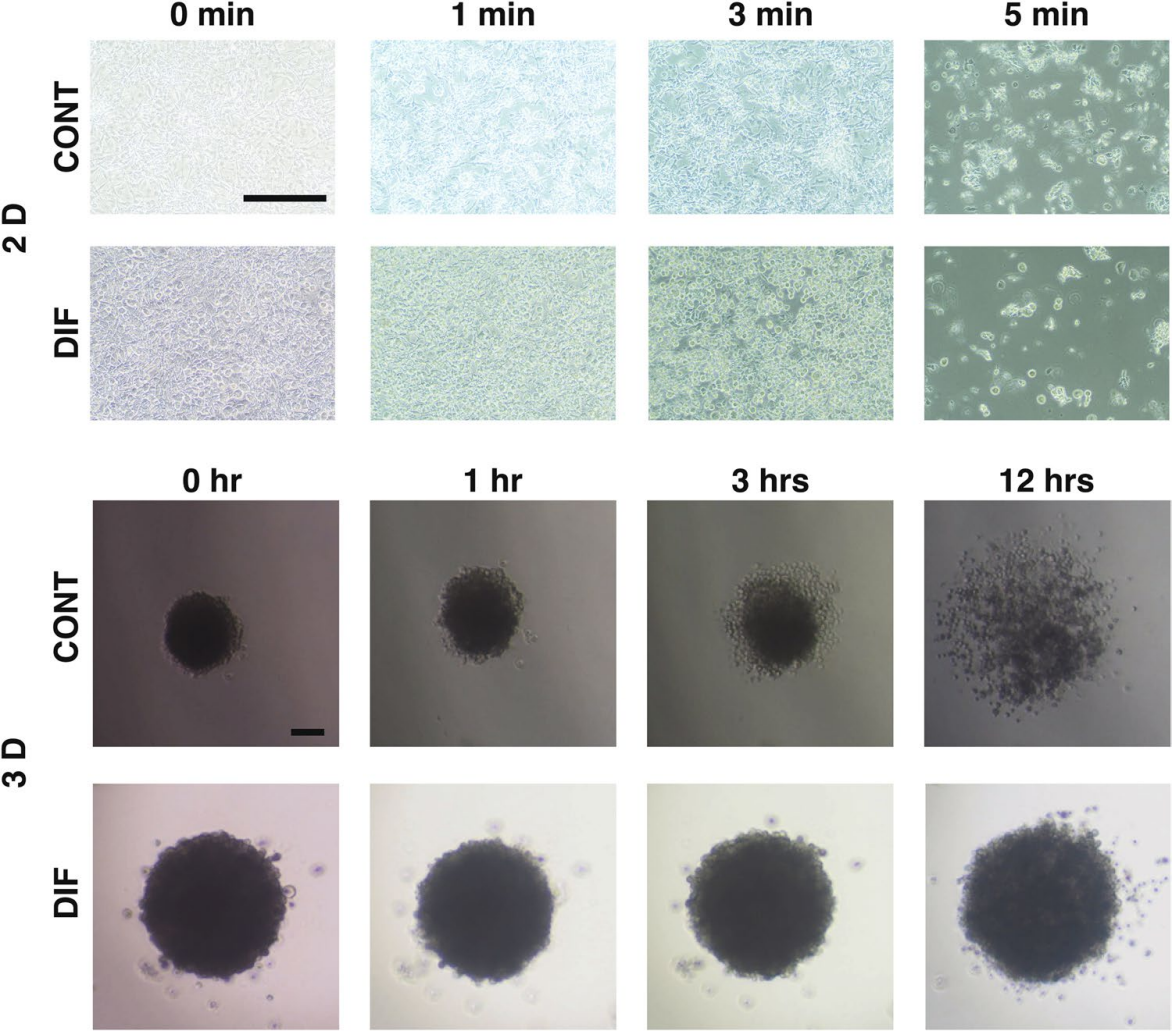
Time course of destruction of formation of 2D colony or 3D organoid of 3T3 L-1 cells by trypsin. To estimate stability of 2D colony and 3D organoid, either CONT or DIF 2D confluent colony or 3D organoids of 3T3 L-1 cells were incubated by 0.2% trypsin and those photos were taken at different time points. All experiments were performed in duplicate using fresh preparations.

However, immunostaining revealed that the staining intensities of COL 1, COL 4, COL 6, and FN significantly decreased through the adipogenesis (Fig. 6A,B). In contrast, such effects by the adipogenesis were less pronounced in all four ECMs by PGs except in the case of COL 4 by BIM-A (Fig. 6A,B). In terms of this discrepancy between mRNA expression and immunostaining intensities, we speculated that spatial localization within the organoids in addition to the abundance of each molecule may have been involved. In fact, to elucidate structural difference between 2D and 3D cell culture from 3T3-L1 cells, trypsin sensitivity was examined (Fig. 7). In 2D cells were rapidly dispersed within few minutes in the presence of 0.2% trypsin, whereas 3D CONT organoid started to disperse after 3hrs and this completed up to 12hrs. Surprisingly, 3D DIF organoids were almost resisted against 0.2% trypsin until 12hrs. Taken together with above observation that lipids generated by adipogenesis surrounded by ECM (Fig. 3A EM and Fig. 6A), these spartial distribution and interaction may be involved to the difference of the trypsin sensitivities.

## Discussion

Concerning the effects of anti-glaucoma medications on adipocytes, Seibold et al^23^. reported that PGs including bimatoprost (BIM), travoprost, latanoprost, and tafluprost suppressed preadipocyte growth. However, other drugs like timolol and benzalkonium chloride didn’t show significant suppression and cytotoxic effects toward adipocytes. Thus, such effects of PGs toward adipocytes were thought to be involved in the etiology of DUES. It is well known that BIM-A is an acid form of prost-type PG analogues. The prost-type PG analogues are derived from PGF2α by the addition of a phenyl base at C-17 and conservation of the C-15 hydroxyl base. And, it is thought that this phenyl base is important for binding to the prostanoid FP receptor^24^. BIM is a prostamide type, and its C-terminal of PGF2α is replaced by ethylamide. In contrast, BIM-A directly binds to a dimer prostamide receptor, which is composed of the FP receptor and an FP receptor splice variant^25^. Based upon these observations, suppression of adipogenesis by PGs may be related to their FP receptors. Recently Taketani *et al*. confirmed that prost-type PG analogues have the effect of inhibiting adipogenesis through FP receptor stimulation using a 2D 3T3-L1 culture and FP receptor knockout mice^24^. In the current study using a 3D cell culture of 3T3L1 cells, we also confirmed mRNA expression of PG FP receptor since early phase of the 3D cell culture (Fig. 2) and furthermore we made the following additional observations: (1) BIM-A and PGF2α significantly suppressed the increase of the organoid sizes, lipid contents, lipid laden structure, and gene expressions of adipogenesis related factors including *Pparγ*, C/EBPα, Ap2 and Adipo Q, and (2) BIM-A and PGF2α affected spatial localization and distribution of ECMs within the 3D organoids during their adipogenetic differentiation.

A 3D organoid culture has been recently used to stimulate a variety of human pathologic conditions^26^. Using a conventional 2D organoid culture technique, evaluations of cell autonomous ECM deposition through synthetic scaffolds such as collagen hydrogels were difficult^27^. The liquid-based droplet spheroid culture employed is better suited for the application. Our group recently established this 3D culture model using human orbital fibroblast cells in the patients with thyroid ophthalmopathy, and found a critical role played by HIF2A in mediating LOX-dependent ECM accumulation^28^. Through evaluating 3T3-L1 organoids, we could better understand the deposition of collagens and FN in a *de novo* ECM meshwork under the adipogenesis condition. In this condition, increased lipid-laden cells were observed and the size of spheroids was significantly larger than that in the preadipocyte condition^29,30^.

ECM have many important roles such as which provides structural support to organs, modifies cell-cell signals, and acceleration or suppression various cellular functions^31^. Collagens (COLs) are triple helical proteins. It existed in the ECM and at the interface between cell and ECM. There are various kind of COLs and COL-related proteins but the most popular COL is COL 1^32^. It is well known that COL 4 is a main component of the basement membrane ECM^33^. COL 6 is one of major ECM that have many functions in different tissues. It is well known that COL 6 has a key role in biomechanical to regulatory signals in the cell survival processes and nervous system. And, COL 6 also plays an important role in determining the differentiation of several types of cells^34^. Fibronectin (FN), which composed of highly interwoven fibers, is present during periods of change within tissues. FN molecules have a weak molecular conformation that can be changed by binding of allosteric partners or strain resulting from cell contractile forces^35^. There are many reports about expressions of COL 1, 4, and 6, and FN in adipocytes or adipose tissues and their modification during adipogenesis were reported^36–38^. It has been reported that a main type of adipose ECM was the main fibril-forming COL 1 and microfibrillar COL 6. The expression of ECM changes characteristically in *in vivo* and in *in vitro* adipogenesis^31^. Moreover, previous studies using a 2D culture of 3T3-L1 preadipocytes^36–38^ revealed *in vitro* remodeling from COL 1- and FN-rich ECM in preadipocyte cells into the further basal membrane type-rich ECM, for example COL 4, in adipocyte cells. In the present study using 3D organoids, down-regulation of COL 1 and FN expressions of 3D 3T3-L1 organoid following differentiation were confirmed as suggested previously as above, not only by their mRNA expressions but also spatial distributions of the molecules within the organoid revealed by immunostaining. Upon adipogenic differentiation, the mRNA expressions of COL 4 and COL 6 also increased in 3D organoids as described previously using 2D cell cultures^39^. Such changes were also confirmed by their immunostaining intensities of 2D cell cultures. In contrast, in the 3D organoids, the immunoreactivities toward COL 4 and COL 6 decreased during the differentiation.

Possible mechanisms causing such difference in the immunoreactivities toward COL 4 and COL 6 between 2D and 3D cultures during adipogenesis have not been elucidated. To get insight into conformational aspects of the 3D organoids of preadipocyte and their adipogenetic conditions, these organoids incubated with 0.2% trypsin. Upon this trypsin exposure, breakdown times of 3D organoids from preadipocyte were much longer than their 2D cell culture. Surprisingly in addition, adipogenetic 3D organoids demonstrated much more resistant against this trypsin treatment (Fig. 7). Based upon these observations, the 3D organoid culture method may provide additional insight into spatial localization of the target molecules within the organoids. Thus, these observations allow us to speculate that PGs might affect not only expression levels of the ECM molecules, but also ECM localization and distribution within the organoids. If this speculation is correct, our new 3D organoid method may be more suitable than the conventional 2D cell culture system for elucidating unknown mechanisms of PG-induced DUES, such as tissue penetration, tissue concentration, affinity for the FP receptor, and activation of the FP receptor. As another possibility, some factors related fibrosis of the organoid such as HIFs, which has been demonstrated as an important factor modulating 3D organoid from thyroid-associated orbitopathy in our recent study, may be related. In our preliminary study, mRNA expressions of HIF2a increased upon adipogenesis of the 3T3-L1 organoid, but those were not significantly affected by the presence of PGs (data not shown). Therefore we will explore other factors involved in this mechanism in our next projects.

In conclusion, our present results suggest that using 3D organoids of 3T3-L1 cells through activation of FP receptors may reduce adipogenesis relevant to clinical DUES. Although the 3T3-L1 is a very novel preadipocyte cell line that can changes adipocytes^40^, we still do not know whether this cell line may be equal to human orbital adipose tissues. As observed in a recent pilot study, similar to the current 3T3L1 organoids, 3D organoids were also successfully obtained from human orbital adipocytes using a similar 3D culture protocol (Supplemental Fig. 1). To clarify the characteristics of human orbital adipogenesis and clinical DUES etiology, as well as to develop means of possible treatment and prevention, further study of primary cultured human orbital adipocytes is needed.

## Materials & Methods

### Chemicals and drugs

Dulbecco’s Modified Eagle’s Medium (DMEM) (# 11965092, Gibco/Thermo Fisher Scientific, Waltham, MA), fetal bovine serum (FBS) (# 16-000-044, Gibco/Thermo Fisher Scientific), L-glutamin (# 25030081, Gibco/Thermo Fisher Scientific), antibiotic/antimycotic (# 15240062, Gibco/Thermo Fisher Scientific), penicillin/streptomycin (# 15140122, Gibco/Thermo Fisher Scientific), Ficoll-Paque Plus (# 17-1440-03, GE Healthcare, Piscataway, NJ), Puromycin (# P8833, Sigma-Aldrich, St Louis, MO), Protamine sulfate salt from salmon (# P4020, Sigma-Aldrich), Methocel A4M (# 94378, Sigma-Aldrich), Dexamethasone (# D1756, Sigma-Aldrich), 3,3’,5-Triiodo-L-thyronine (T3) (# T6397, Sigma-Aldrich), Troglitazone (# 71750, Cayman Chemical, Ann Arbor, MI), Porcine insulin (# I5523, Sigma-Aldrich), Bimatoprost free acid (#16810, funakoshi), Prostaglandin F2α (#16010, funakoshi), AL8810 (#16735, funakoshi).

### Adipocyte culture and differentiation of 3T3-L1 cells

The 3T3-L1 cell (#EC86052701-G0, KAK) is a universally used cell line for lipid studies. The 3T3-L1 preadipocytes were grown until confluence at 37 °C in HG- DMEM containing 8 mg/L d-biotin, 4 mg/L calcium pantothenate, 100 U/mL penicillin, 100 μg/mL streptomycin (b.p. HG-DMEM), and 10% CS.

The 3T3-L1 organoids were generated by a hanging droplet spheroid three dimension (3D) culture system as described recently^28^. Briefly, to facilitate stable morphology, methylcellulose (Methocel A4M) was added to the growth medium. Prior to seeding the hanging drop culture plate (# HDP1385, Sigma-Aldrich), cells were cultured in 100 mm or 150 mm dishes until reaching approximately 90% confluence. After washing with a phosphate buffered saline (PBS), cells were detached using 0.25% Trypsin/EDTA and resuspended in growth medium. After centrifugation for 5 min at 300 g, the cell pellet was re-suspended in a growth medium containing 0.25% w/v Methocel A4M. Volume was adjusted so that 20,000 cells were contained in the 28 μL solution, and 28 μL drops were placed into each well of the drop culture plate (defined as 3D/Day 0). An organoid medium (i.e. growth medium with 0.25% w/v Methocel A4M) was used throughout the duration of the spheroid culture. On every following day, 14 μL of the culture medium was removed and a fresh 14 μL culture medium was added to each well.

### Adipogenic differentiation of organoids, and treatment with anti-glaucoma drugs and/or FR receptor antagonist

Adipogenic differentiation was induced in a medium containing 250 nM dexamethasone, 10 nM T3, 10 μM troglitazone, and 1 μg/ml insulin for two days, and thereafter in a medium with 10 μM troglitazone and 1 μg/ml insulin. To study the influence of several anti-glaucoma drugs, prostaglandin derivatives (PGs) including either 100 nM Bimatoprost free acid (BIM-A), Prostaglandin F2α (PGF2α), latanoprost (LAT), or timolol (Timo) and /or 10 μM AL8810, a specific antagonist of FP receptor were added with the adipogenic differentiation as above.

### Histocytology

To analyze their lipid droplet formation for the 3D culture, the organoids were transferred to 6 super-low attachment well dishes, incubated in 0.2% BODIPY (# D3922, Thermo Fisher Scientific) in PBS for 1 hr, and then fixed in 4% paraformaldehyde (PFA) in PBS for 10 min at room temperature. Fluorescence intensity of the BODIPY-stained lipid droplets was measured using a Nikon A1 confocal microscope (Tokyo, Japan) and quantified using Image J software version 2.0.0 (NIH, Bethesda, MD).

For immunostaining of the 3D culture, organoids prepared as above were fixed in 4% PFA/PBS overnight. After blocking in 3% BSA/0.1% PBS for 3 hrs at room temperature, organoids were washed 3 times for 30 min with PBS. Samples were incubated with a primary antibody including a rabbit anti-collagen monoclonal antibody (collagen 1; # 600-401-103-0.1, collagen 4; # 600-401-106-0.1, or collagen 6; # 600-401-108-0.1, Rockland Immuno-chemicals Inc.) or mouse anti-FN monoclonal antibody (# G0717, Santa Cruz Biotechnology) at 1:200 dilutions overnight at 4 °C. After a subsequent wash in PBS, the organoids were incubated with goat Alexa Fluor 488 anti-rabbit IgG (# A-11070, Thermo Fischer Scientific) or goat Alexa Fluor 594 anti-mouse IgG (# A-11020, Thermo Fischer Scientific) at 1:500 dilutions for 3 hrs at room temperature. Subsequently, for F-actin and nuclear staining, slides were incubated with Alexa Fluor 594 phalloidin (# 20553, Funakoshi) and DAPI (# D523, Dojindo) at 1:1000 dilutions for 3 hrs at room temperature. Slides were then mounted in Prolong Gold before observation by confocal microscope.

### Real-time PCR

Total RNA was extracted from 16 organoids using an RNeasy mini kit (Qiagen, Valencia, CA). Reverse transcription was performed with the SuperScript IV kit (Invitrogen) as per manufacturer’s instructions. Respective gene expression was quantified by real-time PCR with the Universal Taqman Master mix using a StepOnePlus machine (Applied Biosystems/Thermo Fisher Scientific). cDNA quantities were normalized to the expression of housekeeping gene 36B4 (*Rplp0*) and are shown as fold-change relative to the control. Sequences of primers and Taqman probes used are shown in Supplementary Table 1.

### Electron microscopy

The Scanning electron microscopy (EM) specimen was fixed in 4% paraformaldehyde (PFA) in PBS for 1 hour. The specimen was dehydrated through a graded ethanol series and dried at the critical point of CO_2_. The specimen was then mounted on an aluminum stub and coated with osmium using a plasma osmium coater (Nippon Laser and Electronics, Nagoya). The block was viewed under a scanning electron microscope (Hitachi S-4500, Tokyo) operated at 5 keV. The detector features 1280 × 960 pixel.

### Trypsin treatment of 2D cells and 3D organoids of preadipocyte and their adipogenetic conditions

The CONT or DIF 2D confluent colony or 3D organoids of 3T3 L-1 cells were incubated by 0.2% trypsin. Photos of those cells or organoids were taken at different time points (2D cells; 0, 1, 3 or 5 min, 3D organoids; 0, 1 3 or 12 hrs) by using a phase-contrast microscopy (ECLIPSE Ts 2, Nikon, Tokyo).

### Statistical analysis

All statistical analyses were performed using Graph Pad Prism 8 (GraphPad Software, San Diego, CA). For comparison of two mean values, a two-tailed Student’s t-test was used to calculate statistical significance with a confidence level greater than 95%. To analyze the difference in groups, a grouped analysis with two-way analysis of variance (ANOVA) followed by a Tukey’s multiple comparison test was performed. Data are presented as arithmetic means ± standard error of the mean (SEM).

## Supplementary information

Supplemental Dataset.
