# Supplementary material for: Prostaglandin F2α agonist-induced suppression of 3T3-L1 cell adipogenesis affects spatial formation of extra-cellular matrix
Source: Sci Rep. 2020 May 14;10:7958. doi: 10.1038/s41598-020-64674-1 (PMC7224398; doi:10.1038/s41598-020-64674-1)
Supplement: Supplementary file 1 — Supplemental Dataset. [file 41598_2020_64674_MOESM1_ESM.pdf]

Prostaglandin F2α agonist-induced suppression of 3T3-L1 cell adipogenesis affects spatial formation of extra-cellular matrix

Yosuke Ida, Fumihito Hikage, Kaku Itoh, Haruka Ida, Hiroshi Ohguro.

Departments of Ophthalmology, Sapporo Medical University School of Medicine

Supplemental Figure 1. Representative images of phase contrast microscopy of 3D organoids of human orbital adipocytes

Human orbital adipocytes collected as surgical waste were subjected to primary culture and then subsequently subjected to the 3D organoid culture protocol as describe in the current 3T3 L1 cell. Here, the culture period was 12 days rather than 7 days. Scale bar: 100 μm.

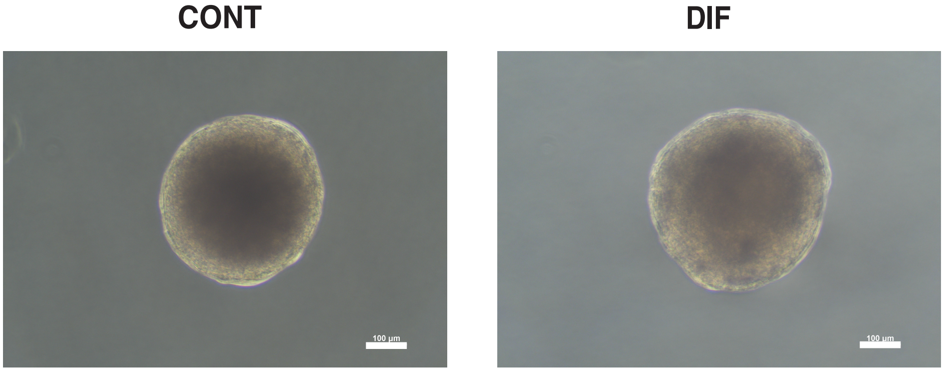

Supplementary Table 1

Sequences of primers and Taqman probes used in the Real-time PCR are shown.

Gene	Forward primer (5' to 3')	Reverse primer (5' to 3')	TaqMan probe (5' to 3')
**Mouse**			
Pparg	CTGCTCCACACTATGAAGACAT	TGCAGGTTCTACTTTGATCGC	/FAM/AGCTGACCC/ZEN/AATGGTTGCTGATTACA/IABkFQ/
Leptin	GTGCCTATCCAGAAAGTCCAG	AATGAAGTCCAAGCCAGTGA	/FAM/ACCGACTGC/ZEN/GTGTGTGAAATGTCA/IABkFQ/
AdipoQ	TGTCTGTACGATTGTCAGTGG	GCAGGATTAAGAGGAACAGGAG	/FAM/ACGACACCA/ZEN/AAAGGGCTCAGGAT/IABkFQ/
C/EBPα	ACAAGAACAGCAACGAGTACC	TCATTGTCACTGGTCAACTCC	/FAM/CGCAAGAGC/ZEN/CGAGATAAAGCCAAAC/IABkFQ/
Ap2	ACTTGTCTCCAGTGAAAACTTTG	ATCACATCCCCATTCACACT	/FAM/CAGGAAAGT/ZEN/GGCTGGCATGGC/IABkFQ/
PGF2receptor	GCCATAATGTGCGTCTCCT	GATCTGATTCCACGTTGCCA	/FAM/TGGAGTCCC/ZEN/TTTCTGGTAACAATGGC/IABkFQ/
Col1a1	CGCAAAGAGTCTACATGTCTAGG	CATTGTGTATGCAGCTGACTTC	/FAM/CCGGAGGTC/ZEN/CACAAAGCTGAACA/IABkFQ/
Col4a1	TCTGGCTGTGGAAAATGTGA	AATCCAATGACACCTTGCAAC	/FAM/TCTTTCTCC/ZEN/CTTTTGTCCCTTCACGC/IABkFQ/
Col6a1	CCAGATGAGTGTGAGATCCTG	AAGTTCTGTAGGCCAATGCTC	/FAM/ACCCATTGA/ZEN/CATCCTCTTCGTGCTG/IABkFQ/
Fn1	GAGCTATCCATTTCACCTTCAGA	TTGTTCGTAGACACTGGAGA	/FAM/CAGGAGATT/ZEN/TGTTAGGACCACGGCA/IABkFQ/
36b4	TTATAACCCTGAAGTGCTCGAC	CGCTTGTACCCATTGATGATG	/FAM/AGGCCCTGC/ZEN/ACTCTCGCTT/IABkFQ/
